# Lung squamous cell carcinoma complicated with immune checkpoint inhibitor-associated active tuberculosis: a case report and literature review

**DOI:** 10.3389/fonc.2026.1848651

**Published:** 2026-09-03

**Authors:** Xiufang Zhang, Yuqiao Zhou, Huiqing Qiu, Zheng Yang

**Affiliations:** 1 Department of Oncology, The Second People’s Hospital of Quzhou, Quzhou, China; 2 Department of Rehabilitative, The Second People’s Hospital of Quzhou, Quzhou, China; 3 Department of Infectious Diseases, The Second People’s Hospital of Quzhou, Quzhou, China

**Keywords:** colorectal cancer, immune checkpoint inhibitors, non-small cell lung cancer, second primary tumor, tuberculosis

## Abstract

Immune checkpoint inhibitors (ICIs) have become one of the standard therapeutic regimens for advanced lung cancer. However, the risk of infection-related complications induced by ICIs, particularly reactivation of tuberculosis, has attracted increasing clinical attention. Herein, we report a 72-year-old male patient with squamous cell carcinoma of the lung who developed reactivation of latent tuberculosis infection (LTBI) approximately 13 months after sequential PD-1 inhibitor therapy. During anti-tuberculosis treatment, the patient was further diagnosed with a second primary ascending colon adenocarcinoma. Combined with this index case and 26 previously published cases of ICI-associated tuberculosis infection identified via literature review, we explore the potential correlation between PD-1/PD-L1 inhibitor therapy and tuberculosis reactivation, as well as corresponding clinical manifestations and comprehensive management strategies. Our findings suggest that clinicians should attach great importance to tuberculosis screening and continuous monitoring throughout the entire course of ICI treatment. For complex patients concurrently complicated with second primary malignancies and infectious diseases, a multidisciplinary team (MDT) approach should be adopted to formulate individualized treatment regimens.

## 1 Introduction

Lung cancer imposes a high global incidence and mortality burden. Immune checkpoint inhibitors targeting the PD-1/PD-L1 axis have emerged as a pivotal therapeutic modality for advanced and locally advanced lung cancer, substantially prolonging patient survival and improving clinical prognosis (1, 2). With the widespread clinical adoption of immunotherapy, immune-related adverse events have been increasingly recognized. Beyond classic immune-mediated inflammatory injuries, infection-associated complications have also garnered growing attention. Among all opportunistic infections, tuberculosis poses a critical safety concern during immunotherapy due to its insidious onset, severe clinical hazards, and profound interference with anti-tumor treatment (3, 4).

Existing studies have demonstrated that the PD-1/PD-L1 pathway exerts a vital regulatory effect on host anti-pathogen immunity and immune homeostasis. Blockade of this pathway may remodel the systemic immune microenvironment, impairing the host’s ability to recognize and clear intracellular pathogens, thereby increasing the risk of opportunistic infections (5, 6). Accumulating clinical evidence indicates that some patients with malignant tumors may develop tuberculosis during PD-1/PD-L1 inhibitor therapy, which falls into three distinct categories: reactivation of LTBI, new primary tuberculosis infection, and tuberculosis reinfection (7). Such infections often present with atypical clinical manifestations, and their radiological features are easily misidentified as immune pneumonitis or tumor progression, which frequently leads to delayed diagnosis and disrupts the overall therapeutic strategy.

On the other hand, the development of second primary malignancies is not uncommon among lung cancer patients with prolonged survival. Nevertheless, cases with concurrent primary lung carcinoma, second primary colorectal carcinoma, and reactivation of LTBI during immunotherapy are extremely rare, posing substantial challenges in treatment timing, regimen selection, risk stratification, and prognosis assessment. Accordingly, this article reports one rare clinical case and reviews relevant published literature to summarize the pathogenesis and clinical characteristics of ICI-associated tuberculosis, and further explore individualized diagnosis, treatment and multidisciplinary management strategies for affected patients, so as to provide clinical references for the management of similar cases.

## 2 Case review

The patient was a 72-year-old Chinese male admitted in June 2024 due to hemoptysis. He had a history of smoking, chronic obstructive pulmonary disease (COPD) and hypertension, while denying prior tuberculosis, malignant tumors or diabetes. No family history of malignancy was recorded. Admission laboratory tests revealed cytokeratin 19 fragment (CYFRA21-1) 11.2 ng/mL and CA125 112.60 U/mL; screening for hepatitis B and human immunodeficiency virus (HIV) yielded negative results.

**Figure 1:**
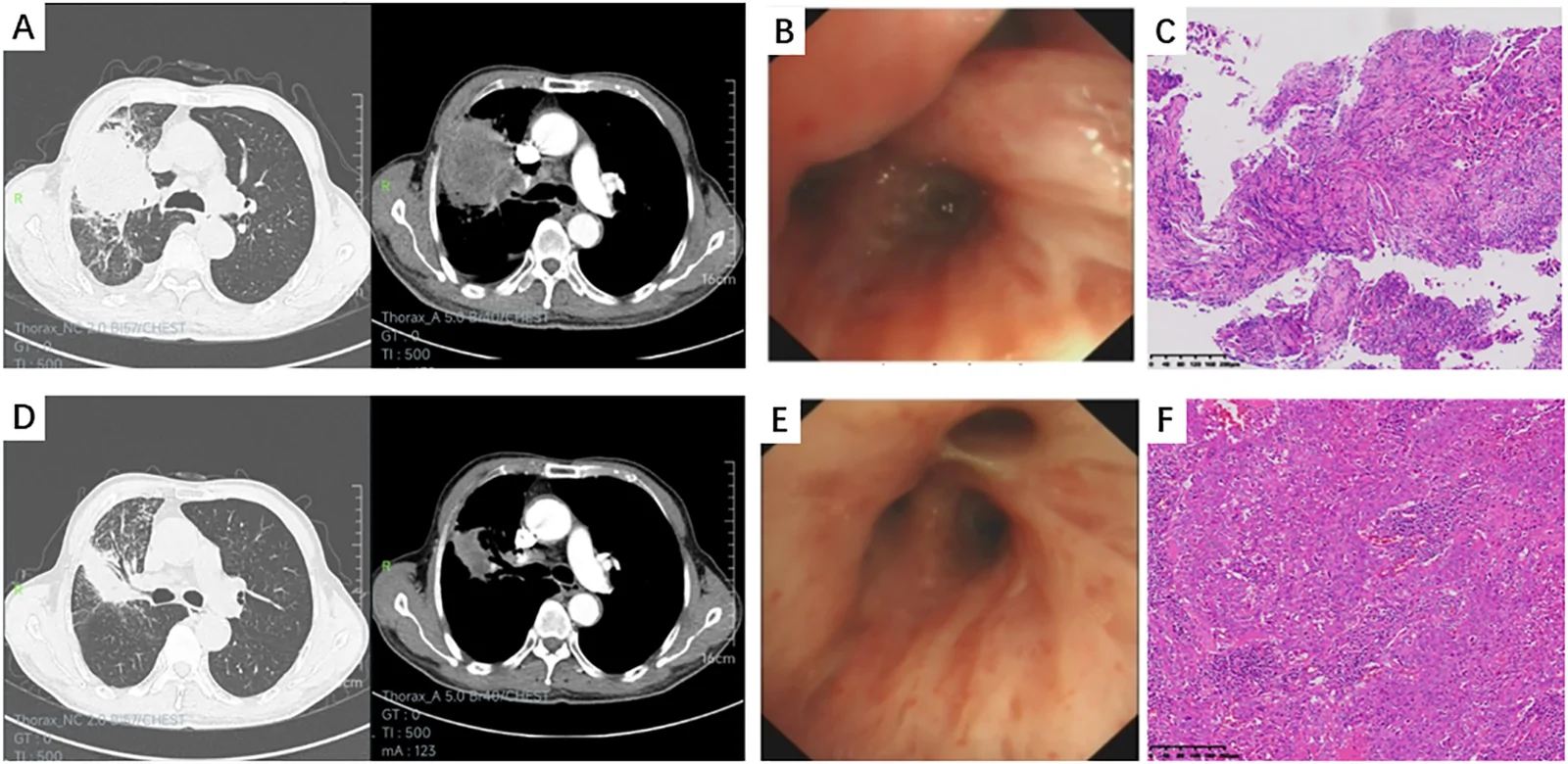
**(A)** Contrast-enhanced chest CT scan in June 2024 showed a soft tissue mass in the right upper lobe, measuring approximately 61mm×65mm, with slight lobulation, spiculated margins, and adjacent bronchial obstruction. The mass exhibited obvious rim enhancement on contrast enhancement. **(B)** Bronchoscopy revealed infiltrative changes in the mucosa of the right upper lobe bronchus, with external compressive stenosis of the lumen. **(C)** Bronchoscopic pathology (HE staining, ×10 magnification) demonstrated squamous cell carcinoma without keratinisation. **(D)** Follow-up contrast-enhanced chest CT scan in October 2024 showed a significant reduction in the size of the right upper lobe mass compared with the previous scan, measuring approximately 37mm×29mm. **(E)** Follow-up bronchoscopy in October 2024 revealed mucosal edema of the right upper lobe bronchus, with a patent lumen. **(F)** Surgical pathology (HE staining, ×10 magnification) confirmed squamous cell carcinoma without keratinisation.

Chest computed tomography (CT) demonstrated a mass in the right upper lobe accompanied by obstructive pneumonia, as well as multiple enlarged mediastinal and right hilar lymph nodes, raising a high clinical suspicion of lung cancer (**Figure 1A**). Bronchoscopy showed mucosal infiltration and extrinsic compression stenosis of the right upper lobe bronchus (**Figure 1B**). Biopsy pathology and immunohistochemistry confirmed squamous cell carcinoma without keratinisation (**Figure 1C**). No distant metastasis was detected on comprehensive staging examinations, conferring a clinical stage of cT3N2bM0, stage IIIB. Neither positron emission tomography-CT (PET-CT) nor endobronchial ultrasound (EBUS) was performed for this patient. Tumor staging was comprehensively determined based on contrast-enhanced chest CT, contrast-enhanced abdominal CT, cranial magnetic resonance imaging (MRI), whole-body superficial ultrasound and bronchoscopic biopsy findings. PD-L1 expression testing and tumor molecular genetic profiling were not carried out in this case.

Prior to the initiation of anti-tumor therapy, the patient underwent a tuberculin skin test (TST) using the Mantoux method, which returned a weakly positive result. According to standard criteria, TST is interpreted as positive at an induration of ≥5 mm in immunocompromised individuals (including patients with malignancy) and ≥10 mm in the general population, indicating underlying LTBI.

No active tuberculous manifestations such as cough, low-grade fever or night sweats were present before treatment, and no active tuberculous lesions were observed on baseline chest imaging. The patient expressed a strong desire for surgical resection and explicitly declined radiotherapy. Preoperative evaluations including cardiopulmonary function, pulmonary ventilation reserve, blood gas analysis and cardiac function were completed. The patient had an Eastern Cooperative Oncology Group (ECOG) performance status of 0 with well-controlled underlying comorbidities. An MDT consisting of thoracic surgery, medical oncology, radiology and respiratory medicine conducted joint consultation. Combined with findings from the phase II TRAILBLAZE trial (8) and the Chinese Expert Consensus on Perioperative Immunotherapy for Non-Small Cell Lung Cancer (NSCLC), the MDT concluded that the patient could tolerate neoadjuvant chemoimmunotherapy followed by radical surgery without absolute contraindications.

The patient received four cycles of neoadjuvant therapy with nab-paclitaxel/carboplatin plus sintilimab from June to September 2024. Reassessment demonstrated remarkable shrinkage of the pulmonary mass and lymphadenopathy (**Figure 1D**), and repeat bronchoscopy revealed a patent right upper lobe bronchial lumen (**Figure 1E**), indicating a partial response (PR). Video-assisted thoracoscopic surgery (VATS) right upper lobectomy plus mediastinal lymph node dissection was performed in October 2024. Postoperative pathology showed major pathological response (MPR) after neoadjuvant therapy with residual viable tumor less than 10%, and all resected lymph nodes were free of metastasis. The pathological stage was ypT2bN0M0, stage IIA (**Figure 1F**). Postoperatively, four cycles of adjuvant chemotherapy with paclitaxel/carboplatin plus tislelizumab were administered, followed by six cycles of single-agent tislelizumab maintenance therapy.

**Figure 2:**
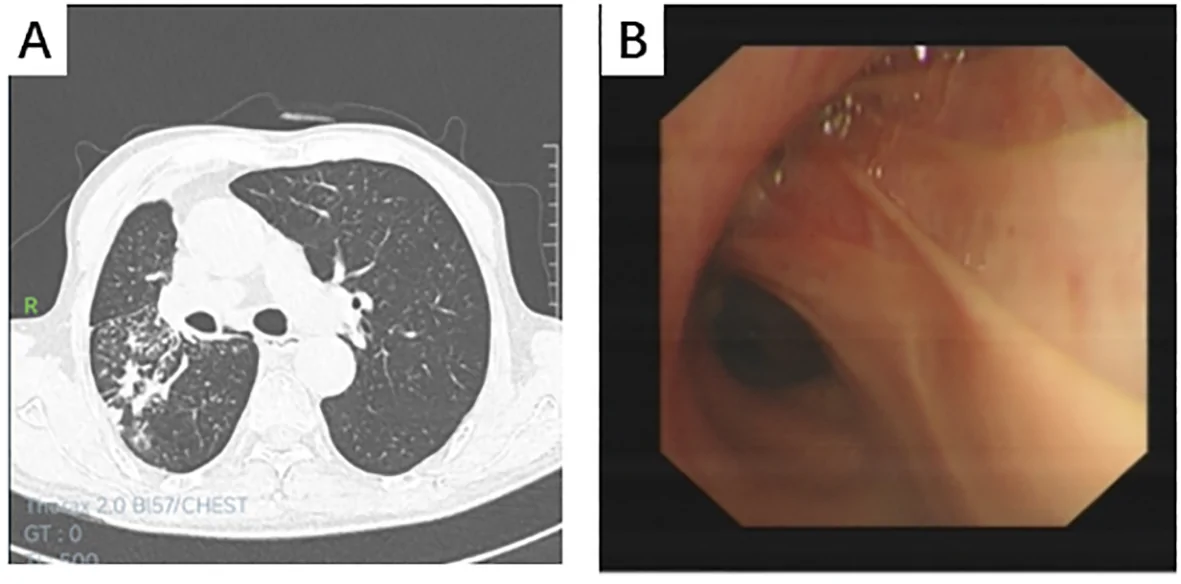
**(A)** Chest CT scan in July 2025, showed multiple bilateral pulmonary infiltrates and ground-glass opacities, accompanied by right pleural thickening. **(B)** Bronchoscopy revealed the stump of the right upper lobe bronchus; the mucosa of the remaining lobar and segmental bronchi was smooth with patent lumens. Bronchoalveolar lavage (BAL) was performed at the dorsal segment of the right lower lobe.

A follow-up chest CT performed in July 2025 revealed progressive bilateral pulmonary infiltrates and ground-glass opacities (**Figure 2A**). Bronchoscopy examination visualized the bronchial stump of the right upper lobe, with smooth mucosa and patent lumens in all other lobar and segmental bronchi (**Figure 2B**). Both Xpert MTB/RIF assay and mycobacterial culture of bronchoalveolar lavage (BAL) fluid tested positive for *Mycobacterium tuberculosis* complex with rifampicin-susceptible strain. Combined with the patient’s weakly positive baseline TST result, active pulmonary tuberculosis caused by reactivation of LTBI was confirmed. Standard anti-tuberculosis treatment with the 2HRZE/4HR regimen was initiated in July 2025.

**Figure 3:**
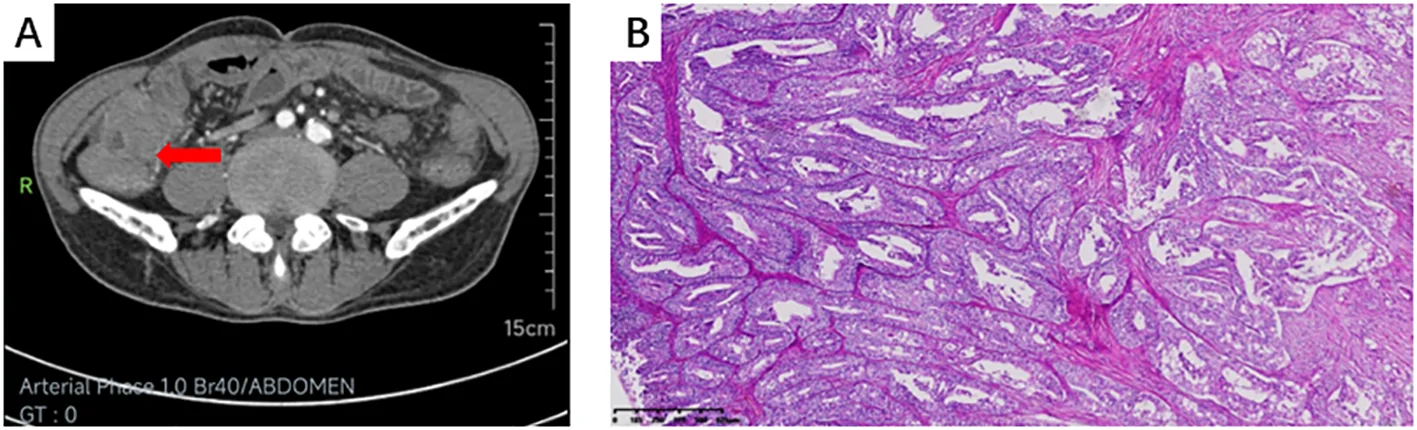
**(A)** Contrast-enhanced whole-abdominal CT scan in September 2025, showed focal thickening of the ileocecal intestinal wall. Neoplastic lesion was considered the primary diagnosis, and the possibility of intestinal tuberculosis could not be excluded in combination with the patient’s medical history. **(B)** Postoperative pathology (HE staining, ×10 magnification) revealed moderately differentiated adenocarcinoma of the ascending colon, with the lesion invading the serosal layer.

During anti-tuberculosis treatment, the patient developed recurrent nausea, vomiting, and abdominal distension. Anti-TB medications were temporarily discontinued, and further investigations were performed. Contrast-enhanced whole-abdominal CT scan suggested thickening of the ileocecal intestinal wall, with the possibility of tumor or intestinal tuberculosis not excluded (**Figure 3A**). In September 2025, the patient underwent laparoscopic right hemicolectomy + adhesiolysis. Postoperative pathology revealed moderately differentiated adenocarcinoma of the ascending colon, staged as pT3N0M0, stage IIA, with all lymph nodes showing reactive hyperplasia (**Figure 3B**).

After MDT consultation and evaluation, chemotherapy and immunotherapy were suspended. The patient was in the consolidation phase of anti-tuberculosis treatment, and anti-tumor therapy including immunotherapy might exacerbate immune suppression and hinder tuberculosis control.

Liver metastases were identified on follow-up imaging in December 2025, and the primary origin of the metastatic lesions could not be differentiated from lung squamous cell carcinoma or colorectal adenocarcinoma. Meanwhile, the patient suffered severe acute intracerebral hemorrhage and was admitted to the intensive care unit (ICU) in critical condition. He was too critically ill to tolerate percutaneous needle biopsy or surgical tissue sampling, so the primary source of liver metastases could not be further confirmed. Despite aggressive supportive treatment, the patient’s condition did not improve. The patient’s family ultimately opted to discontinue active treatment, and the patient succumbed to the disease within a short period.

## 3 Results of literature review

### 3.1 Literature search strategy and selection criteria

We conducted an independent systematic literature search using PubMed. The search period spanned January 2016 to January 2026 to collect English original articles and case reports concerning ICI-associated tuberculosis among patients with malignancies. Search terms: (“PD-1 inhibitor” OR “PD-L1 inhibitor” OR “immune checkpoint inhibitor”) AND (tuberculosis OR mycobacterium tuberculosis OR latent tuberculosis reactivation) AND (cancer OR carcinoma OR tumor).

Inclusion criteria: (1) patients with confirmed malignancies treated with single or combined ICI therapy; (2) microbiologically or pathologically confirmed active tuberculosis (new infection, latent reactivation or reinfection) occurring during or after ICI administration; (3) accessible complete clinical data on ICI regimens, tuberculosis onset, clinical manifestations, management and prognosis; (4) English publications with available full texts.

Exclusion criteria: (1) tuberculosis diagnosed before ICI commencement; (2) cases with missing key clinical data or duplicate publications; (3) conference abstracts and non-English articles; (4) cases of nontuberculous mycobacterial infections.

Literatures were initially screened by titles and abstracts, followed by full-text evaluation. Duplicates and ineligible records were excluded per the above criteria. Ultimately, 26 published eligible cases were retrieved. Together with the index case in the present study, 27 cases were included for pooled descriptive analysis (**Table 1**).

**Table 1:** Summary analysis of clinical and literature cases.

Study objects	Age/gender/nationality	Cancer types	Combination therapy	ICIs	Time lag (months)	Radiologic features of TB	Clinical manifestations of TB	Diagnosis of TB	Tuberculous lesion	Treatment of TB	discontinue ICI therapy	restart ICIs	TB infection classification	Outcomes
Lee (9)	87/M/Chinese	Hodgkin lymphoma	Chemo + RT	Pembrolizumab, 5 cycles	4	No abnormal findings on chest CT	Fever, weight loss	Sputum culture (+)	Pulmonary TB	Isoniazid, Rifampicin, Ethambutol	Yes	No	Reactivation of LTBI	Lymphoma remission; TB stable
Fujita 2016 (10)	72/M/Japanese	Lung squamous cell carcinoma	Chemotherapy	Nivolumab, 8 cycles	4	New centrilobular nodules with tree-in-bud sign in bilateral lungs	No specific TB-related symptoms	BAL culture (+), PCR (+), IGRA conversion from negative to positive	Pulmonary TB	Specific medication is not specified	Yes	No	New primary infection	NR
Chu 2017 (11)	59/M/Chinese, Taiwan	Lung adenocarcinoma	Gefitinib + chemotherapy	Nivolumab, 3 cycles	3	Rapid pericardial effusion, pericardial thickening	Cardiac tamponade	Pericardial fluid culture (+), pericardial histology	Tuberculous pericarditis	Specific medication is not specified	Yes	Yes	Reactivation of LTBI	Effusions resolved; no recurrence after ICI
Takata 2019 (12)	75/M/Japanese	Lung adenocarcinoma	Chemo + RT	Nivolumab, 15 cycles	10	New pulmonary opacities in right middle and lower lobes	Fever, cough, purulent sputum	Sputum AFB (+), PCR (+), sputum culture (+)	Pulmonary TB	HREZ	Yes	Yes	Unclassifiable	TB cured; tumor PR; paradoxical reaction relieved
Anastasopoulou 2019 (13)	76/F/ Greek	Cutaneous melanoma	IFN + steroids + infliximab	Nivolumab ± ipilimumab, 8 cycles	6	New GGOs and centrilobular nodules in bilateral lungs (predominantly right)	Fever, fatigue, weight loss, lower respiratory tract infection	BAL culture (+), PCR (+)	Pulmonary TB	HREZ	Yes	No	New primary infection	Respiratory deterioration; death
Anastasopoulou 2019 (13)	85/M/ Greek	Parotid melanoma	IFN + cobimetinib	Atezolizumab	5	No new abnormalities on initial chest X-ray	Lower respiratory tract infection, fever	Sputum culture (+)	Pulmonary TB	HREZ	Yes	Yes	Unclassifiable	On ATT, well-tolerated; antitumor therapy suspended
Barber 2019 (14)	59/M/ American	Nasopharyngeal carcinoma	None	Nivolumab, 3 cycles	1	Diffuse GGOs and centrilobular nodules in bilateral lungs	No specific TB-related symptoms	Lung AFB (+), PCR (+), histology; BAL & stool culture (+)	Disseminated TB (lung, rectum, intestine/peritoneum)	HREZ initially; streptomycin, moxifloxacin, linezolid for intestinal perforation	Yes	No	Reactivation of LTBI	Death
Barber 2019 (14)	83/M/ American	Metastatic Merkel cell carcinoma	None	Pembrolizumab, 11 cycles	6	New 1.1×1.6 cm nodule in right lower lobe	No specific TB-related symptoms	Lung AFB (+), histology;	Pulmonary TB	HREZ, Levofloxacin, Rifapentine were administered after Isoniazid administration	Yes	Yes	Reactivation of LTBI	TB cured; tumor responded to ICI
Sirgiovanni 2021 (15)	44/M/ German	SCLC	Chemo + RT	Nivolumab + ipilimumab, 3 cycles	2	New lesions in bilateral upper lobes, cavitation in left upper lobe	No specific TB-related symptoms	BAL AFB (+), PCR (+); sputum & gastric juice AFB (+)	Pulmonary TB	HREZ	Yes	No	New primary infection	Lesions reduced; tumor stable; 24-month disease control
Kato 2020 (16)	75/F/ Japanese	NSCLC	Chemo + RT	Durvalumab, 5 cycles	2	Diffuse bilateral infiltrates, cavitary primary lesion enlargement, right middle/lower lobe tree-in-bud sign	Intermittent fever (38–39°C), night sweats	Sputum AFB (+), PCR (+); T-SPOT.TB (+)	Pulmonary TB	Specific medication is not specified	Yes	No	Reinfection	TB controlled; tumor stable
Lau 2021 (17)	29/F/ Chinese, Hong Kong	Nasopharyngeal carcinoma	Chemo + RT + steroids	pembrolizumab	20	PET-CT: terminal ileitis, enlarged mesenteric lymph nodes	Abdominal pain, vomiting, mucoid bloody stools, persistent high fever	Ileal PCR (+), histology; QFT-IGRA (+); AFB (−)	Terminal ileum, mesenteric lymph nodes	HREZ,	Yes	Yes	Reactivation of LTBI	TB cured; tumor shrank after ICI
Suliman 2021 (7)	58/F/ Qatari	Lung adenocarcinoma	None	Pembrolizumab, 6 cycles	12	Left lung consolidation and cavitation; subsequent diffuse reticulonodular infiltrates bilaterally	Cough, sputum, high fever, tachycardia, hypoxemia	BAL AFB (+), PCR (+); sputum AFB (+)	Pulmonary TB	HREZ	Yes	No	Reactivation of LTBI	Sputum negative; tumor near-CR
Murakami 2021 (18)	73/M/ Japanese	Lung adenocarcinoma	Steroids	Pembrolizumab, 5 cycles	18	Right lower lobe faint opacity → GGO + nodule; right upper lobe consolidation + nodule	Initially asymptomatic, later cough/sputum without fever	Sputum culture (+), PCR (+); sputum AFB (−)	Pulmonary TB	HREZ	Yes	Yes	Unclassifiable	TB lesions resolved; tumor PR; no TB relapse
Riudavets 2022 (19)	42/M/ French	Pulmonary sarcoma	Chemo + RT	Pembrolizumab, ~30 cycles	32	Peritoneal infiltration, enlarged mesenteric/retroperitoneal lymph nodes with necrosis	Night sweats, fever, no typical respiratory symptoms	Retroperitoneal lymph node histology; clinically suspected TB	disseminated TB: Peritoneum, mesentery, retroperitoneal lymph nodes, multiple bones	HREZ	Yes	No	Unclassifiable	Symptoms relieved; tumor CR; TB controlled
He 2018 (20)	65/M/ Chinese	Malignant melanoma	High-dose IL-2	Pembrolizumab, 10 cycles	8	Right upper lobe posterior segment opacity	Hemoptysis for 1 week	BAL GeneXpert (+), culture (+); lung histology	Pulmonary TB	HREZ	Yes	Yes	Reactivation of LTBI	Tumor CR; TB cured without reactivation
Jensen 2018 (21)	56/M/ Caucasian	Lung adenocarcinoma	Chemo + RT	Nivolumab, 12 cycles	3	Progression of right upper lobe anterior segment infiltration with cavitation; new infiltration at right apex	Progression of right upper lobe anterior segment infiltration with cavitation; new infiltration at right apex	Lung histology, AFB (+); surgical specimen PCR (+)	Pulmonary TB	Specific medication is not specified	Yes	No	Reactivation of LTBI	ATT initiated; no malignancy; tumor stable
Picchi 2017 (22)	50/M/ French	Malignant melanoma	NR	Pembrolizumab, 4 cycles	3	NR	Asymptomatic pleurisy	Pleural histology	Pleura	The “four-drug” regimen	No	No	Unclassifiable	Pleural effusion resolved
Picchi 2017 (22)	64/M/ French	Metastatic NSCLC	NR	Nivolumab, 2 cycles	1	NR	Spinal cord compression	Bone culture (+), PCR (+), histology	Bone	The “four-drug” regimen	Yes	No	Unclassifiable	Rapid death after spinal surgery
Nikola 2023 (23)	52/F/ Serbian	Lung adenocarcinoma	Chemotherapy	Pembrolizumab, 33 cycles	25	Multiple nodules, micronodules, tree-in-bud sign, GGOs in bilateral upper lobes	Fever, dry cough, weight loss	BAL AFB (+), culture (+), PCR (+)	Pulmonary TB	HREZ	Yes	No	New primary infection	Temporary improvement; death from liver failure
Tsai 2019 (24)	49/M/ Chinese, Taiwan	Hard palate squamous cell carcinoma	Chemo + RT	Nivolumab	3	Patchy high density, consolidation, and cavitary mass-like lesion in left upper lung	Fever, purulent sputum for 2 weeks, bilateral crackles	Sputum AFB (+), culture (+), PCR (+)	Pulmonary TB	Specific medication is not specified	Yes	No	Unclassifiable	Diarrhea after ICI; death from tumor progression
Van 2019 (25)	56/F/Swiss	Lung adenocarcinoma	Chemo + RT + steroids	Nivolumab	12	New pulmonary nodules, right lung infiltration, lymphangitis carcinomatosa	Dyspnea, cough, weight loss	Sputum AFB (+)	Pulmonary TB	HREZ	Yes	Yes	Reactivation of LTBI	Diarrhea after ICI; death from tumor progression
Elkington 2018 (26)	62/F/ British	Uveal melanoma	None	1. Ipilimumab 2. Pembrolizumab; stable for ~2 years	24	Apical cavitary lesion	No specific TB-related symptoms	BAL culture (+); liver & lung histology	Disseminated TB (lung, liver)	Specific medication is not specified	Yes	No	Unclassifiable	Symptoms improved; lesions regressed
Lin 2023 (27)	68/M/ Chinese	Lung squamous cell carcinoma	Chemotherapy	Camrelizumab, 1 cycle	1	Increased multiple small nodules, patchy opacities and infiltrates in bilateral lungs	Cough, sputum, chest distress, dyspnea, hemoptysis	BAL PCR (+), AFB (+)	Pulmonary TB	HREZ	Yes	No	New primary infection	TB lesions resolved; tumor stable
Tetikkurt 2018 (28)	53/M/ Turkish	Maxillary squamous cell carcinoma	Chemo + RT	Pembrolizumab, 6 cycles	4	Chest CT consistent with TB infection	No specific TB-related symptoms	PCR (+); typical radiologic features of TB	Pulmonary TB	Specific medication is not specified	Yes	Yes	Reactivation of LTBI	TB controlled; tumor shrank after ICI
Hirano 2024 (29)	67/M/ Japanese	Lung adenocarcinoma	Chemotherapy	Nivolumab + ipilimumab	1	Initial: cavity near primary lesion in right upper lung; recurrence: diffuse left lung infiltration + right upper lung cavity	Initial: increased sputum; recurrence: anorexia, impaired consciousness	Sputum culture (+), AFB (+), PCR (+); identical strain by VNTR	Pulmonary TB	Initial: HREZ; Recurrence: Isoniazid, Rifampicin, Levofloxacin, Streptomycin	Yes	Yes	Reactivation of LTBI	Sputum negative; TB recurrence; death from respiratory failure
Suo 2023 (30)	44/M/ Chinese	Esophageal squamous cell carcinoma	Chemo + RT	Tislelizumab, 15 cycles	10	Multiple reversed halo signs and consolidations in bilateral lungs	Low-grade fever, cough, non-purulent sputum, exertional dyspnea, hypoxemia	BAL MTB detected by NGS	Pulmonary TB	HREZ	Yes	No	Unclassifiable	Lesions resolved; symptoms improved
Yang 2025	72/M/ Chinese	Lung squamous cell carcinoma	Chemotherapy	1.Sintilimab, 4 cycles 2.tislelizumab, 10 cycles	13	Bilateral pulmonary inflammation, increased right lung lesions	No specific TB-related symptoms	BAL Xpert MTB/RIF (+), culture (+)	Pulmonary TB	2HREZ/4HR	Yes	No	Reactivation of LTBI	On ATT; liver metastasis; sudden death from cerebral hemorrhage

### 3.2 Summary of the 27 cases

Analysis of the 27 cases of ICI-associated tuberculosis infection included in this study (including the present index case) revealed that the cohort consisted mainly of middle-aged and elderly patients (age range, 29–87 years; median, 62 years), with a male predominance (20/27, 74.1%). NSCLC constituted the most common primary malignancy (13/27, 48.1%), followed by melanoma (5/27, 18.5%), head and neck squamous cell carcinoma (3/27, 11.1%), and others. This distribution pattern is influenced by multiple confounding factors: NSCLC represents the major approved indication for ICIs with an enormous treated patient population; smoking serves as a shared risk factor for both lung cancer and tuberculosis. In addition, tuberculosis and NSCLC each inherently exhibit higher incidence among males, which also contributes to the male predominance observed in this cohort. Owing to the absence of aggregate data on the total number of patients receiving ICIs across different tumor types, comparative analyses of infection risks among various patient subgroups could not be performed. The immune agents administered in these cases were predominantly PD-1 inhibitors (pembrolizumab in 11 cases, nivolumab in 11 cases, and others in 5 cases). The median time-lag from ICI initiation to active tuberculosis diagnosis was 5 months (IQR, 3-12 months; range, 1-32 months). The number of patients diagnosed within 12 months after ICI initiation was adjusted to 19/27 (70.4%) in the revised manuscript.

Most patients presented with non-specific symptoms rather than classic tuberculous manifestations. Fever was observed in 11 of 27 patients (40.7%), and cough in 7 of 27 patients (25.9%). Eight patients (29.6%) were asymptomatic, with tuberculous lesions incidentally detected by imaging. Pulmonary imaging manifestations were heterogeneous and easily misdiagnosed as immune-related pneumonitis or tumor progression; nodules, consolidations or infiltrates represented the most frequent imaging pattern (19/27, 70.4%). Tree-in-bud sign, ground-glass opacities, and cavitary lesions were also observed. Definitive diagnosis relied on positive acid-fast bacilli smears, nucleic acid detection, Xpert MTB/RIF assays, and mycobacterial cultures from sputum, bronchoalveolar lavage fluid, or lesion tissues. Pathological biopsy confirming granulomatous inflammation or caseous necrosis was documented in 10 cases (37.0%). Tuberculosis involving the lung occurred in 22 of 27 patients (81.5%). Disseminated tuberculosis involving multiple organs occurred in 3 patients, while isolated extrapulmonary tuberculosis was seen in 4 additional patients. Regarding infection classification, reactivation of latent tuberculosis infection (LTBI) accounted for 12 cases (44.4%), new primary infection for 5 cases (18.5%), reinfection for 1 case (3.7%), and 9 cases (33.3%) could not be definitively classified.

The standard four-drug anti-tuberculosis regimen (isoniazid, rifampicin, pyrazinamide, ethambutol) was widely adopted (19/27, 70.4%). Regarding ICI adjustment, 26 patients (96.3%) discontinued ICI after the diagnosis of active tuberculosis; among them, 10 patients (37.0%) resumed ICI after adequate tuberculosis control. Current clinical evidence suggests that ICI should be promptly discontinued once active tuberculosis is confirmed to initiate standardized anti-tuberculosis therapy. After effective tuberculosis control, carefully selected patients may cautiously resume ICI under close monitoring. There is no unified consensus regarding the optimal timing for ICI re-initiation; an interval of 2-4 weeks is generally recommended in clinical practice (13). Patient outcomes were heterogeneous: 14 patients (51.9%) achieved tuberculosis control or cure with stable tumor status, whereas 8 patients (29.6%) died due to tumor progression, severe adverse drug reactions, multi-organ involvement, or severe complicated infection.

Collectively, ICI-associated tuberculosis is characterized by insidious onset, atypical manifestations, high misdiagnosis rates and complex clinical management, which necessitates constant clinical vigilance and individualized comprehensive management throughout the entire treatment course.

## 4 Discussion

The widespread application of immune checkpoint inhibitors, particularly PD-1/PD-L1 inhibitors, has significantly improved the prognosis of lung cancer patients, yet it has also brought a series of previously underrecognized immune-related adverse events, among which secondary tuberculosis stands out as an unignorable clinical safety concern (3). The PD-1/PD-L1 pathway exerts unique bidirectional immunoregulatory functions: it suppresses T cell activation to mediate tumor immune escape, and simultaneously upregulates PD-1 expression on CD4^+^ T cells within latent tuberculous granulomas to restrain excessive Th1 cell activation and exaggerated secretion of pro-inflammatory cytokines including IFN-γ and TNF-α, thereby maintaining granuloma structural integrity. This pathway serves as a critical protective mechanism for the host to contain latent tuberculosis and sustain immune homeostasis (31, 32). ICIs block the PD-1/PD-L1 axis to reverse T cell exhaustion and restore T cell activity for enhanced anti-tumor immunity (33). However, such blockade ablates the negative immune regulatory mechanism within tuberculous lesions, triggering aberrant activation of tuberculosis-specific CD4^+^ Th1 cells and amplified type I interferon signaling. This inflammatory cascade triggers an exaggerated immune response that disrupts granulomatous structure, promotes massive release and dissemination of dormant *Mycobacterium tuberculosis*, and ultimately leads to tuberculosis reactivation (34, 35). This pathological process essentially represents inflammatory injury driven by excessive immune reconstitution, which is distinct from opportunistic infections secondary to conventional immunosuppressive agents and bears striking clinical similarities to human immunodeficiency virus-associated immune reconstitution inflammatory syndrome (IRIS) (36). PD-1 knockout animal models further validate that loss of PD-1 function exacerbates pulmonary inflammatory infiltration, tissue necrosis and elevated mycobacterial burden following tuberculosis infection, with uncontrolled Th1 immune responses as the core driver of rapid disease deterioration (37). Furthermore, intrinsic tumor-related immune dysregulation, ICI therapy, and auxiliary medications used to manage immune-related adverse events can further disrupt immune balance and elevate the risk of LTBI reactivation (38, 39).

From the perspective of clinical therapeutic strategies, rifampicin, a core component of the standard 2HRZE/4HR anti-tuberculosis regimen, is a potent cytochrome P450 3A4 (CYP3A4) inducer. It upregulates metabolic enzyme activity via pregnane X receptor activation, which can reduce the plasma concentrations of CYP3A4 substrate drugs by up to 95%, and this inductive effect lasts for several weeks (40]. Taxane chemotherapeutics and epidermal growth factor receptor tyrosine kinase inhibitors (EGFR-TKIs) (e.g., SH-1028, gefitinib) are typical CYP3A4 substrates. Co-administration with rifampicin drastically diminishes systemic exposure to anti-tumor agents and impairs therapeutic efficacy (40–42). Accordingly, clinicians must remain highly alert to drug-drug interactions in patients co-affected by malignancy and tuberculosis, avoid concurrent administration of the aforementioned anti-tumor drugs with rifampicin whenever possible, and implement individualized dose adjustments guided by therapeutic drug monitoring if necessary.

The case reported in this study exhibits distinctive clinical features. The 72-year-old male patient was diagnosed with stage IIIB squamous cell carcinoma of the right lung. He achieved PR after neoadjuvant chemoimmunotherapy with nab-paclitaxel, carboplatin and sintilimab, followed by adjuvant chemoimmunotherapy combining paclitaxel, carboplatin and tislelizumab plus subsequent single-agent tislelizumab maintenance. Approximately 13 months after immunotherapy initiation, LTBI reactivated and progressed to active pulmonary tuberculosis. After standard 2HRZE/4HR anti-tuberculosis treatment was commenced, recurrent nausea and vomiting prompted imaging identification of ileocecal wall thickening. Subsequent surgery confirmed moderately differentiated adenocarcinoma of the ascending colon at stage IIA, representing an extremely rare clinical scenario complicated by synchronous primary lung squamous cell carcinoma, second primary colorectal adenocarcinoma, and reactivation of LTBI under ICI treatment.

Although second primary malignancies are not uncommon among lung cancer survivors, cases simultaneously complicated by primary lung squamous carcinoma, second primary colorectal cancer, and reactivated LTBI during ICI treatment remain rarely documented domestically and internationally. Such cases pose multiple therapeutic conflicts, including mutual constraints between anti-tuberculosis therapy, postoperative adjuvant lung cancer treatment, and curative colorectal cancer resection. After comprehensive multidisciplinary evaluation, immunotherapy was permanently discontinued in this patient, with priority assigned to tuberculosis control followed by colorectal tumor resection. This clinical decision embodies the core principle of prioritizing infection control while balancing multi-tumor management in patients with complex comorbidities, and aligns with the central conclusion drawn from our literature review: patients developing active tuberculosis during immunotherapy require early recognition and timely initiation of standardized anti-tuberculosis treatment, with individualized regimens formulated based on tumor control status, tuberculosis severity and overall patient performance status.

In summary, based on the pooled case data and existing literature evidence, we strongly recommend universal LTBI screening for all patients prior to PD-1/PD-L1 inhibitor administration, particularly in regions with high burdens of lung cancer, lymphoma and tuberculosis, where screening should be established as a routine mandatory procedure. During immunotherapy, any new pulmonary infiltrates or unexplained fever warrant routine tuberculosis differential diagnosis, rather than presumptive attribution to immune pneumonitis or tumor progression. PD-1/PD-L1 inhibitor therapy elevates the risk of active secondary tuberculosis in lung cancer patients; such infections present with concealed clinical manifestations, atypical imaging features and high management complexity, with additional therapeutic challenges superimposed by concurrent second primary malignancies. Clinical practice should enforce standardized pre-treatment tuberculosis screening, continuous on-treatment surveillance and multidisciplinary collaborative workflows. The core management strategy of early identification, prioritized anti-tuberculosis intervention and individualized anti-tumor therapy should be followed to improve diagnostic and therapeutic proficiency for such complex cases and optimize patient prognosis.

This study has several limitations. Our literature search was limited to the PubMed database only, which may introduce selection bias. In addition, the small sample size restricts the statistical reliability of our findings. As this work combines retrospective case analysis with a literature review, incomplete clinical records for some retrieved cases preclude accurate classification of the specific subtype of active tuberculosis infection.

## 5 Conclusions

PD-1/PD-L1 inhibitor therapy increases the risk of secondary active pulmonary tuberculosis in lung cancer patients. Such infections present with occult clinical manifestations and non-specific imaging features that are readily confused with immune pneumonitis or tumor progression, highlighting the critical necessity of early recognition and standardized anti-tuberculosis intervention. The present case represents an extremely rare coexistence of primary lung squamous cell carcinoma, second primary ascending colon adenocarcinoma and reactivated LTBI triggered by immunotherapy. This case highlights that patients receiving ICIs face not only elevated infectious risks but also potential synchronous second primary malignancies, which substantially complicate clinical decision-making. All patients scheduled for ICI treatment should undergo pre-treatment infectious disease screening and close longitudinal monitoring throughout therapy; timely tuberculosis-related assessments are indicated for new pulmonary lesions or unexplained fever. For patients with multiple concurrent diseases, a multidisciplinary collaborative approach should be adopted, prioritizing infection control and designing individualized comprehensive therapeutic regimens tailored to tumor status. This strategy maximizes treatment safety, minimizes adverse events and improves overall patient prognosis.

## Data Availability

The original contributions presented in the study are included in the article/supplementary material. Further inquiries can be directed to the corresponding author.
